# Two-Color Infrared Sensor on the PbTe: In *p-n* Junction

**DOI:** 10.3390/s21041195

**Published:** 2021-02-08

**Authors:** Jonas Gradauskas, Bohdan Dzundza, Leonid Chernyak, Zinovy Dashevsky

**Affiliations:** 1 Department of Electronic Processes, Center for Physical Sciences and Technology, 10257 Vilnius, Lithuania; 2 Department of Physics, Vilnius Gediminas Technical University, 10223 Vilnius, Lithuania; 3 Department of Computer Engineering and Electronics, Vasyl Stefanyk Precarpathian National University, 76018 Ivano-Frankivsk, Ukraine; bohdan.dzundza@pnu.edu.ua; 4 Department of Physics, University of Central Florida, Orlando, FL 32816-2385, USA; Leonid.Chernyak@ucf.edu; 5 Department of Materials Engineering, Ben-Gurion University of the Negev, 84105 Beer-Sheva, Israel; dashevsky.45@mail.ru

**Keywords:** infrared sensor, high-temperature PbTe photodiode, specific detectivity, two-photon absorption

## Abstract

A lead telluride sensor was fabricated on the base of a p-n PbTe junction created on a PbTe single crystal grown by the Czochralski technique, followed by the diffusion of an indium donor impurity into a crystal. The capacitance-voltage and current-voltage characteristics of the sensor were measured over the temperature range from 80 K to 150 K. A prototype of a high-temperature mid-IR sensor, a PbTe diode, with a cut-off wavelength of 4 μm, operating at temperatures up to 150 K, was demonstrated for the first time. The advantage of the sensor is that its operating temperature is high enough to be reached by a solid-state thermoelectric cooler. The sensor showed a specific detectivity value of 10^10^ cm Hz^1/2^/W at a temperature of 150 K and a wavelength of 4.2 μm. The possibility to sense pulses of long-IR radiation by means of the PbTe diode was also demonstrated over the 100–180 K temperature range. For the first time, a two-photon absorption-caused photovoltaic effect was observed in PbTe at a wavelength of 9.5 μm at 150 K.

## 1. Introduction

Photodiodes based on InSb compounds are traditional semiconductor sensors for detection in the midinfrared region because of their forbidden energy gap *E*_g_ close to 200 meV at operating temperature *T* = 77 K (cooled by liquid nitrogen) and demonstrated a specific detectivity D* of about 10^11^ cm Hz^1/2^/W [1,2,3].

On the other hand, the PbTe semiconductor compound, having a practically comparable value of *E*_g_ = 210 meV (*T* = 77 K), is also used for the fabrication of IR sensors [4,5]. The advantage of PbTe in comparison with InSb is the increase of its energy gap with growing operating temperatures. Thus, it is possible to use PbTe photodiodes up to a cooling temperature of 180 K [6]. Therefore, practically for the first time, it has become possible to cool these sensors with solid-state coolers based on multistage thermoelectric modules.

Photodiode infrared sensors can be either of the Schottky barrier [7] or of the *p-n* junction type. PbTe photodiodes are usually made from layers grown on BaF_2_ substrates [8] or on Si substrates using fluoride buffer layers [9,10,11].

In this work, we fabricated an indium-doped PbTe *p-n* junction on a single crystal grown by the Czochralski technique [12] and investigated its electrical and detection characteristics. The estimated parameters of the IR sensor demonstrate its high photodetection performance: D* = 1.5 × 10^10^ cm Hz^1/2^/W at temperature *T* = 150 K and radiation wavelength λ = 4.2 μm. For the first time, a two-photon absorption-caused photovoltaic effect was observed in PbTe at a wavelength of 9.5 μm at *T* = 150 K.

As mentioned, in contrast to classical semiconductors such as Si or GaAs, the bandgap *E*_g_ of lead telluride increases with the temperature varying from 190 meV at 0 K to 306 meV at 300 K [4] as

(1) $$E_{g}\left(\;T\right)\;=\;0.19\;+\;{4.5\;\times \;10}^{-4}\;T^{2}/\left(T+50\right)$$

**Figure 1:**
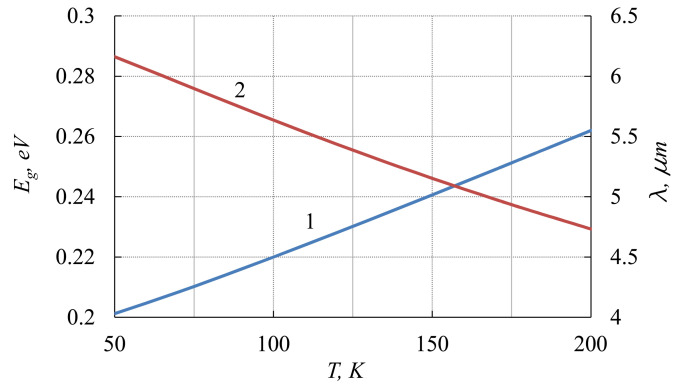
Bandgap of PbTe (1) and the cut-off wavelength (2) as functions of temperature.

Correspondingly, the cut-off wavelength of PbTe calculated as λ = 1.24/*E*_g_(eV) μm as a function of temperature is shown in Figure 1.

## 2. Detector Fabrication

The single crystals of the PbTe semiconductor were grown by means of the Czochralski method, which allows unidirectional solidification from the melt [12]. The synthesized PbTe was loaded into a quartz crucible, which was heated in a furnace to 1200 °C. A PbTe seed crystal of <100> orientation was used to pull the crystal out of the melt. A molten boron trioxide B_2_O_3_ layer about 1 cm thick was used for liquid encapsulation. The molten boron trioxide was inert to PbTe at the solidification temperature. The temperature gradient at the crystallization was 25 K cm^−1^, and the crystal pull rate was 10 mm h^−1^. The crystal and the crucible were rotated in opposite directions at an angular velocity of 1 s^−1^.

The crystal orientation was determined by the Laue method. The Cu_Kα1_ radiation (α = 1.5406 Å, Δ2Ѳ = 0.005°, 2Ѳ range 10–120°) and Bragg-Brentano geometry were employed in an X-ray diffractometer. The Hall effect measurements were carried out in a permanent magnetic field *B* = 2 T with an accuracy of 8%.

To form the *p-n* junction, one face of the p-type PbTe single crystal was polished, and then an oxide layer was grown electrochemically [13]. The required pattern was formed by ordinary photolithography. Then, the diffusion of the In dopant was carried out from the In_4_Te_3_ gaseous phase into the PbTe crystal [14]. In this process, the PbTe crystal was placed in a quartz ampoule, evacuated to a residual pressure of 10^−5^ mbar, and then heated in a furnace up to 500 °C. The diffusion time was 10 h. The surface indium concentration was determined by the energy-dispersive X-ray spectroscopy (EDS) method. As a result, the *p-n* diodes with respective hole and electron densities *p* ≈ 10^18^ cm^−3^ and *n* = 2 × 10^18^ cm^−3^ (*T* = 80 K) were produced.

## 3. Device Properties

### 3.1. Electrical Characteristics

**Figure 2:**
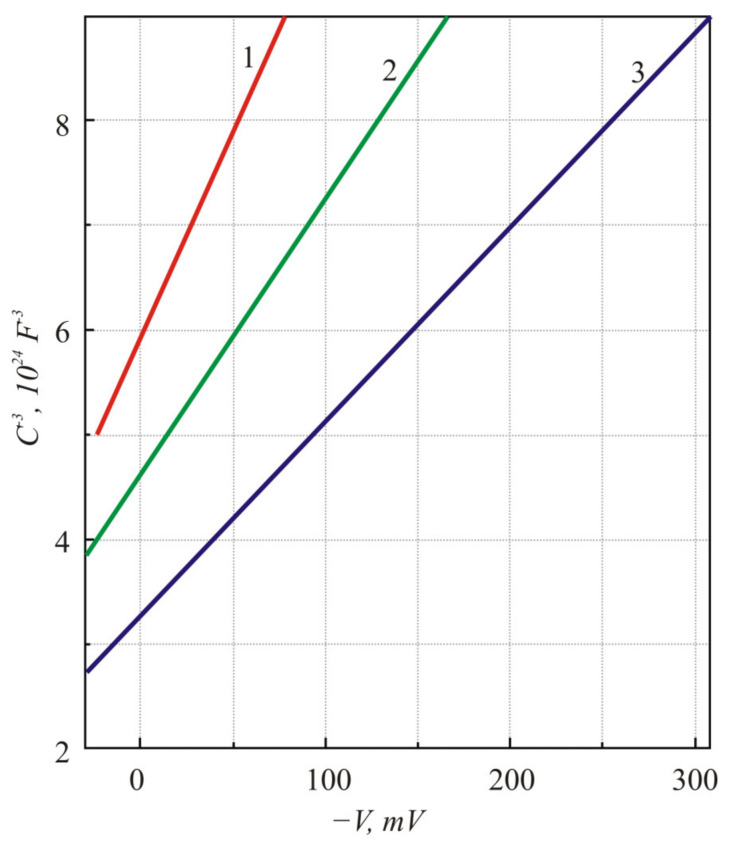
Capacitance-voltage characteristics of the PbTe *p-n* junction at different temperatures: 1—150 K; 2—110 K; 3—80 K.

The capacitance versus voltage, *C-V,* characteristic of the In-doped PbTe *p-n* junctions was measured over a temperature range of 80–150 K with an Agilent HP4280A (Agilent Technologies, Santa Clara, US) capacitance meter [6]. The diodes were placed in a He gas closed-cycle cryostat in a vacuum of about 10^−7^ Torr. The capacitance was measured through an alternate signal of 10 or 30 mV at a frequency of 1 MHz applied along with the DC voltage. The fine linearity of *C*^−3^ curves as a function of voltage *V* agrees well with the standard theory of a linearly graded *p-n* junction [15] (Figure 2).

**Figure 3:**
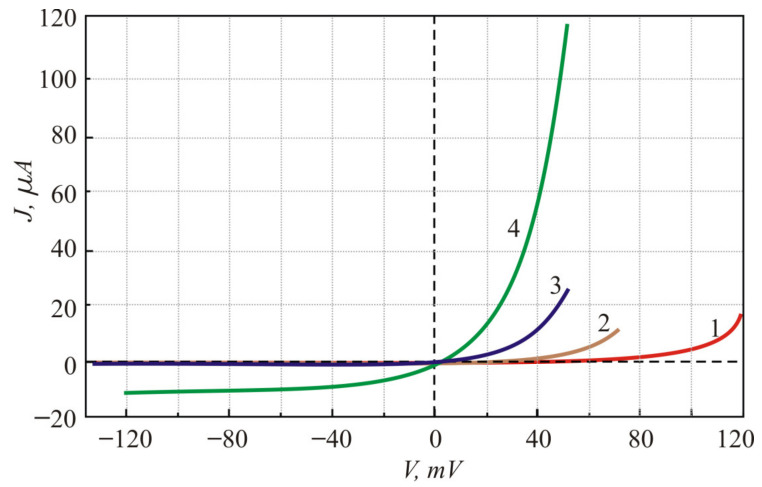
Current–voltage characteristics of the In-doped PbTe photodiode at different temperatures: 1—80 K; 2—100 K; 3—120 K; 4—150 K.

The experimental *I-V* curves of the PbTe *p-n* structure are presented in Figure 3. These curves have been fitted by the Shockley equation [15]:

(2) $${I\;=\;I}_{o}[\exp(eV/nkT)-1]$$

where *I_o_* is the saturation current, and *n* is the ideality factor. This way, the ideality factor was evaluated to equal 1.7, 1.6, 1.7 and 1.8 at, respectively, 80 K, 100 K, 120 K and 150 K temperatures [6]. It is worth noting that, in spite of the inverse dependence of *E*_g_ on temperature, both the reverse current and the forward turn-on voltage of the PbTe *p-n* diodes depend on temperature in the same manner typical of diodes of the classical semiconductors, such as Si, Ge, or GaAs.

### 3.2. Sensing Characteristics

**Figure 4:**
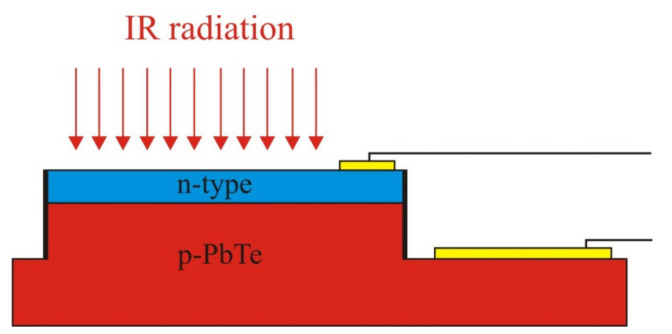
Schematic view of the In-doped PbTe *p-n* photodiode.

Detectivity of the In-doped PbTe *p-n* junction was measured at 150 K in the infrared range following the earlier described techniques [8]. A schematic view of the detector is presented in Figure 4.

The radiation from a blackbody source (*T* = 1000 K) chopped at a 1000 Hz frequency was directed to the detector through a BaF_2_ window in a He gas closed-cycle cryostat. The radiation intensity was *I*_R_ = 10^−3^ W/cm^2^, and the root-mean-square voltage signal *V*_rms_ was measured by means of a lock-in amplifier (Tektonix Differential Preamplifier ADA400A, Shangahi, China) with a bandwidth Δ*f* = 10 Hz. The detector background noise *V*_n_ was measured at 300 K and a field of view equal to 25°. The integral detectivity D* was calculated as [8]

(3) $$\;D*\;=\;\left(\frac{1}{I_{R}}\right)\sqrt{\frac{\Delta f}{A}}\frac{V_{rms}}{V_{n}}$$

where *A* = 1 × 1 mm^2^ is the active area of the detector.

**Figure 5:**
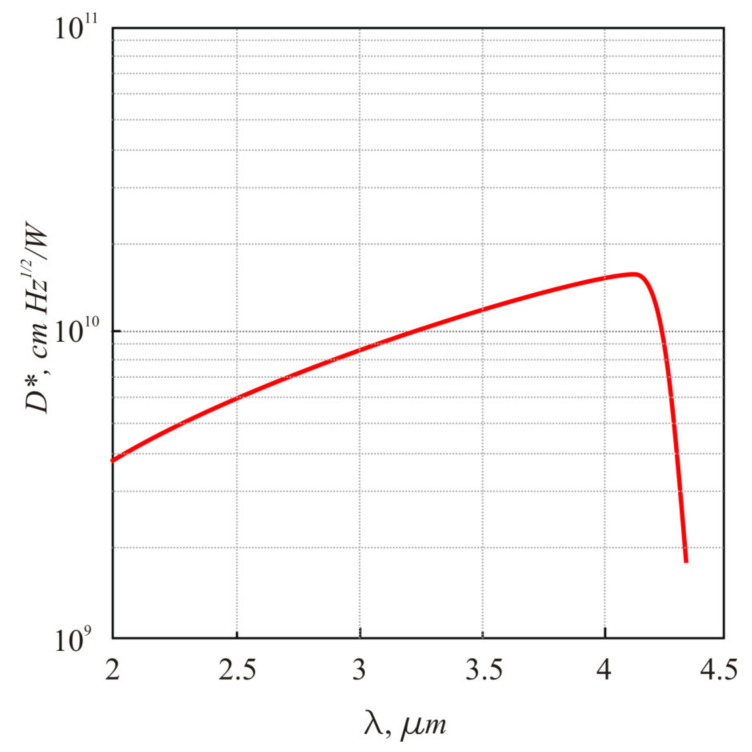
Spectral response of the In-doped PbTe *p-n* junction at *T* = 150 K.

The value of D* ≈ 10^10^ cm Hz^1/2^/W was obtained for the PbTe photodiode at 150 K. The spectral response was measured in the same setup by placing a grating monochromator between the infrared source and the PbTe detector. Figure 5 presents the spectral response characteristic of the In-doped PbTe *p-n* junction at *T* = 150 K. The cut-off wavelength ≈4.2 μm corresponds to the PbTe energy gap *E*_g_ at *T* = 150 K.

The *R*_o_*A* values taken from [6] allowed estimating the specific detectivity *D*_λ_* of the detector. It was defined as [6]

(4) $$D_{\lambda }*\;=\;\frac{R_{\lambda }}{{\left[\frac{4kT}{R_{o}A_{e}}+2e^{2}\eta Q_{B}\right]}^{\frac{1}{2}}}$$

(5) $$R_{\lambda }=\eta e/hc$$

(6) $$Q_{B}\left(\nu_{c},T\;\right)\;=\;J\;\left(\nu \right)d\nu$$

(7) $$J\left(\nu \right)=\frac{8\pi \nu^{2}}{2c^{2}\left[\exp\left(\frac{h\nu }{kT}\right)-1\right]}$$

Here *R_λ_*, is the current mode responsivity for the wavelength *λ*, *η* is the quantum efficiency, and *ν_c_* is the cut-off frequency. For blackbody radiation at 300 K, typical values are *η* = 0.5 and *R*_λ_ ≈ 1.6 A/W at a cut-off wavelength of 4 μm [6]. The estimated value of the specific detectivity *D*_λ_* was 2.2 × 10^10^ cm Hz^1/2^/W at a temperature of 150 K.

As a source of long-wavelength IR radiation, a Q-switched CO_2_ laser operating at a 9.5 μm wavelength with a pulse duration of 150 ns, a repetition rate of 40 Hz, and a maximum intensity of about 0.1 MW/cm^2^ was used. The measurements were performed in the photocurrent regime, i.e., the photovoltage across a 50 Ohm load resistance was recorded by a LeCroy Wavepro 7200 (LeCroy Corporation, Chestnut Ridge, US) oscilloscope with a 2 GHz passband. The measurements were carried out in the same closed-cycle optical cryostat.

**Figure 6:**
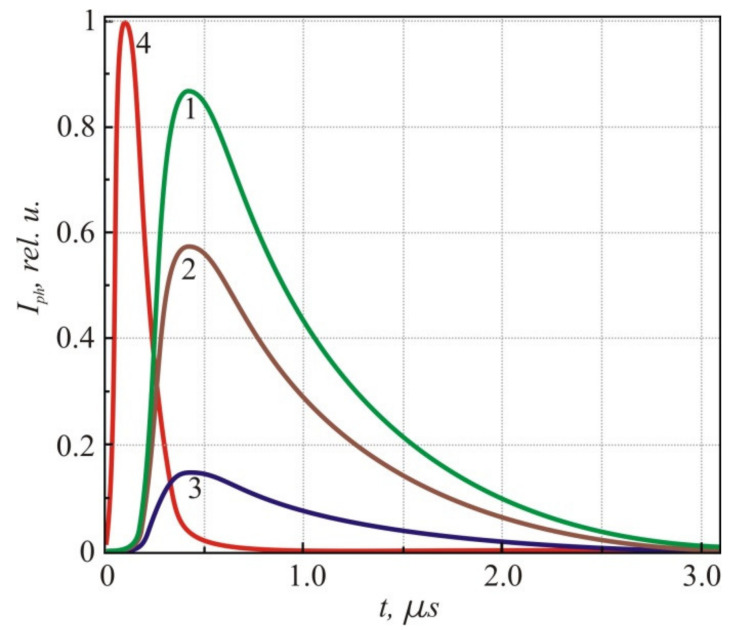
Photocurrent pulses across the PbTe *p-n* junction at different temperatures: 1—100 K; 2—150 K; 3—180 K. For comparison in the timescale, Trace 4 (red) represents the laser pulse.

The fabricated PbTe *p-n* junction sensed the CO_2_ laser radiation. The photocurrent demonstrated a polarity similar to that of the classical one originating from the electron-hole pair generation. Figure 6 presents the dependence of the photocurrent pulses induced by the CO_2_ laser across the *p-n* PbTe structure as a function of time in the 100–180 K temperature range.

**Figure 7:**
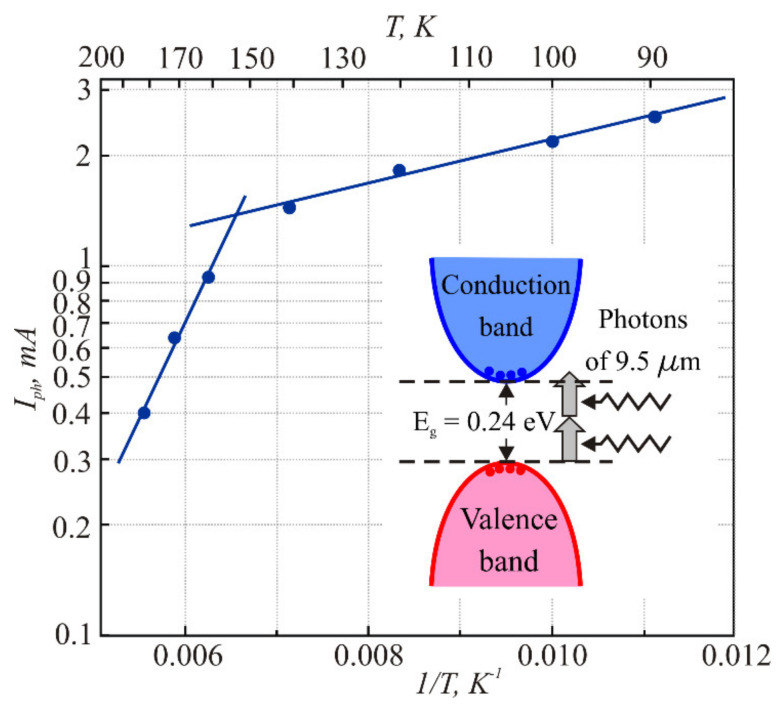
Dependence of the photocurrent across the PbTe *p-n* junction on temperature. Straight lines are guides for the eye of exponential ranges. In inset: schematic view of the two-photon absorption in PbTe at 150 K.

It can be seen that the photocurrent is stronger at lower temperatures. The responsivity of the sensor to 9.5 μm radiation was 10^−6^ A/W at a 150 K temperature. The typical photocurrent pulse relaxation time was about 6 × 10^−7^ s. It did not depend on the temperature within the given range; thus, its origin cannot be attributed to the thermal effects, which, like thermal conductivity, should depend on the sample temperature. The dependence of the peak value of the CO_2_ laser-induced photocurrent pulse on the diode temperature is shown in Figure 7. Two linear slopes can be distinguished in the photocurrent dependence on reciprocal temperature (Figure 7). These indicate the exponential dependence of the photocurrent *I_ph_*:

(8) $$I_{ph}\propto \exp\left(\frac{E_{a}}{kT}\right)$$

where *E*_a_ is the specific activation energy. One of the slopes discloses an activation energy of 100 meV, which corresponds to half of the PbTe forbidden energy gap, i.e.,

(9) $$I_{ph}\propto \exp\left(\frac{E_{g}}{2kT}\right)$$

This finding supports our assumption that the photovoltage should result from the interband generation of electron and hole pairs. The CO_2_ laser photon energy (130 meV) is lower than the forbidden energy gap of PbTe of 216–253 meV over the entire investigated temperature range of 100–180 K, respectively. Therefore, the only mechanism of such generation is two-photon absorption [16]. The inset in Figure 7 illustrates the scheme of the two-photon absorption process. The increase of the photocurrent peak value with lower temperatures is related to the temperature dependence of the forbidden energy gap of PbTe: the lower the temperature, the narrower the energy gap and, correspondingly, the stronger the two-photon absorption.

Another activation energy is equal to 12 meV. The question of this absorption mechanism remains open. It may be related to the impurity levels of indium *E*_In_ that at low temperatures are located in the conduction band close to its bottom [17]. It was shown that the indium doping of PbTe leads to the formation of long-lived electronic states. However, it is still not clear whether these states are formed by the impurity atoms or whether the doping causes the appearance of another type of defect forming quasilocal states. The model for the formation of defective states in PbTe and its alloys was considered in Refs. [18,19,20,21,22,23,24]. Indium impurity has a variable valency and produces a DX center, not a trap [19]. When an electron from lower state E_2_ absorbs a long-wave photon, it moves into another energetic state E_1_ on the In level and thus into another configuration coordinate (with respect to space and area) [18]. In this case, the long-term relaxation effect of photoconductivity appears due to the formation of an effective barrier caused by the change of two units in the impurity valency upon photoexcitation [18,19]. The two-electron nature of the ground state of the center is confirmed by the absence of a paramagnetic resonance signal of the defects. In this case, the energy level of the defect according to [18] and the corresponding analytical expression for the energy of the center that has captured *n* electrons (*n* = 0, 1, or 2) is

(10) $$\varepsilon_{n}=\frac{\Delta^{2}}{{2\Delta }_{o}}+\left(\varepsilon_{o}-\Delta \right)n+U_{n}$$

where *Δ* is the coordinate of the configuration determined in energy units, and *Δ* is equal to the energy shift of one-electron defect level; *Δ*_o_ determines the strength of the electron-phonon interaction in the center, and *ε*_o_ is the energy position of the empty level of the center. *U_n_* = *U* × *β* is the energy of the Coulomb repulsion of the two electrons in the center, and *β* is the Kronecker symbol [18]. The expression for *U* is [18]:

(11) $$U\approx \frac{e^{2}}{\varepsilon r}\approx 10\;\mathrm{meV}$$

Here, *ε* is the permittivity of PbTe [6], and *r* ≈ 1 nm is the radius of the quasilocalized electronic state.

Therefore, the optical absorption with activation energy *E*_a_ ≈ 12 meV can be associated with electron transition from the low energy level *E*_2_ to the higher level *E*_1_ [18].

**Figure 8:**
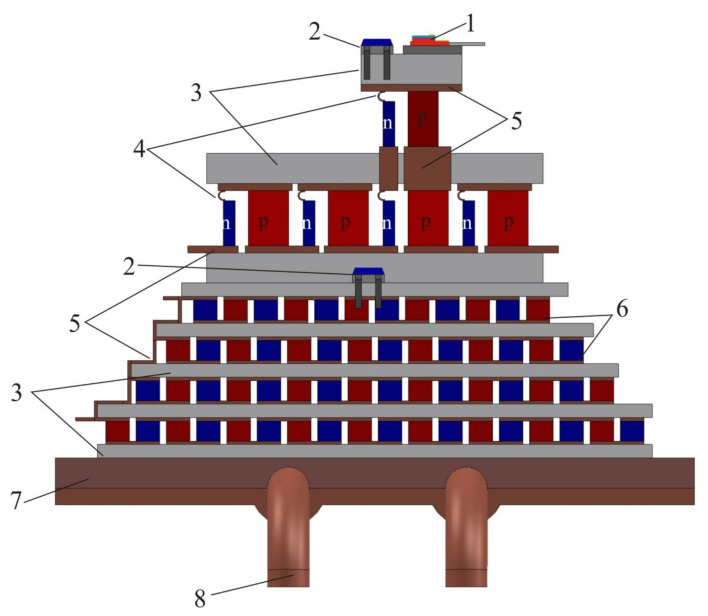
Schematic view of the multistage thermo electric cooler (MTEC). 1—IR detector with the In-doped PbTe *p-n* junction; 2—platinum thermistors; 3—BeO_2_ ceramic; 4—copper foil tape; 5—copper busbars; 6—thermocouples of a multistage thermoelectric module; 7—heat sink; 8—heat pipe.

The authors of Ref. [24] have developed a multistage thermoelectric cooler maintaining a temperature of 140–150 K on the cold side. A schematic view of this cooler is presented in Figure 8. The heat sink (7 in Figure 8) is highly efficient if based on heat pipes since the designed power is about 100 watts (*I* = 5.1–6.4 A, *U* = 12–14 V, electric power *P* = 60–90 W). Such heat sinks are quite light due to their very thin fins, and the heat pipes allow placing the sensor in a convenient place. The first part of the cooler is a four-stage module based on the BiTe compound and operates within the 300–180 K temperature range. The second low-temperature two-stage part of the cooler uses *n*-BiSb and *p*-BiSbTe alloys and operates at the 180–140 K temperature range, i.e., below 150 K.

## 4. Conclusions

The PbTe *p-n* junction diode was fabricated on a PbTe single crystal grown by the Czochralski technique and followed the diffusion of an indium donor impurity into the crystal. The capacitance-voltage and current-voltage characteristics of the sensor were measured over the temperature range from 80 K to 150 K. The saturation current density was ~10^−5^ A/cm^2^ at *T* = 90 K, while at *T* = 150 K it was ~10^−1^ A/cm^2^.

The concept of an IR sensor on the base of a PbTe diode with a cut-off wavelength *λ*_c_ ≈ 4 μm operating at temperatures up to 150 K was demonstrated for the first time. The estimated parameters of the IR sensor demonstrate its high photodetection performance: *R*_o_*A* = 1.5 Ω cm^2^, *J*_o_ = 3 × 10^−3^ A/cm^2^, and D* ≈ 10^10^ cm Hz^1/2^/W at temperature *T* = 150 K and wavelength *λ* ≈ 4.2 μm.

The possibility to sense pulses of long-IR radiation with the PbTe diode was also demonstrated over the 100–180 K temperature range. For the first time, a two-photon absorption-caused photovoltaic effect was observed in PbTe for the wavelength *λ* = 9.5 μm at *T* = 150 K.

The advantage of the new diode lies behind its successful operation at temperatures higher than the cryogenic ones, i.e., above 120 K, which opens the perspective of its application supported (cooled) by a solid-state thermoelectric refrigerator.
